# NAD+ improved experimental autoimmune encephalomyelitis by regulating SIRT1 to inhibit PI3K/Akt/mTOR signaling pathway

**DOI:** 10.18632/aging.203781

**Published:** 2021-12-20

**Authors:** Jinli Wang, Xueqin Song, Guojun Tan, Pengtao Sun, Li Guo, Ning Zhang, Jueqiong Wang, Bin Li

**Affiliations:** 1 Department of Neurology, The Second Hospital of Hebei Medical University, Shijiazhuang 050051, Hebei, China; 2 Department of Neurology, The Second Hospital of Hebei Medical University, Key Laboratory of Hebei Neurology, Shijiazhuang 050051, Hebei, China

**Keywords:** NAD+, SIRT1, thymic epithelial cells, thymus autophagy, immunoregulation, bioinformatics

## Abstract

Objective: To investigate the effect of NAD+ on thymus autophagy in experimental autoimmune encephalomyelitis (EAE) mice through SIRT1.

Methods: Bioinformatic analysis was used to identify hub genes. Forty female C57BL/6 mice were randomly divided into 4 groups: control, EAE, NAD+, and NAD+ +SIRT1 inhibitor (SIRT-IN-3) groups and SIRT1 group. The NAD+ group and SIRT1 inhibitor group were treated with NAD+ drug and fed for 4 weeks. The neurological function scores were evaluated weekly. The thymus tissues of wild-type mice were removed, ground and filtered into single-cell suspension. MOG 35-55 (1 μg/mL) was given to primary thymic epithelial cells (TECs) to induce EAE model *in vitro*. The expression of LC-3A/B was observed by immunofluorescence. The expressions or the activation/phosphorylation of associated proteins were detected by Western blot.

Results: Enrichment analysis showed PI3K-Akt-mTOR and autophagy pathway were main terms in EAE diseases, and the relationship between NAD+ and SIRT1. The activation of p-PI3K, p-Akt and p-mTOR were the highest in the EAE group consistent with decreased P62, Beclin1, LC-3A/B and SIRT1, and NAD+ reversed these results, furthermore SIRT1 inhibitor: SIRT-IN3 weakened the NAD+’ effects in both *in vivo* and *in vitro* experiments. Immunofluorescence study *in vivo* and *in vitro* were accord with the results of western blot.

Conclusions: NAD+ exerted a protective effect on EAE mice by inhibiting PI3K/Akt/mTOR signaling pathway through SIRT1 in TECs, and prevented EAE mice from sustained damage.

## INTRODUCTION

Multiple sclerosis (MS) is an autoimmune inflammatory demyelinating disease of the central nervous system (CNS), which is pathologically characterized by chronic inflammatory demyelinating, reactive proliferation of astrocytes and axon damage to varying degrees. Most commonly occur in women aged between 20 and 40, and their clinical manifestations are visual impairment, ataxia, walking difficulties, paresthesia, sexual dysfunction, urinary and feces disorders, as well as anxiety, depression, insomnia and other psychiatric symptoms [1, 2]. The mechanisms of MS were generally believed to be the auto-immune response for myelin antigen induced by T lymphocytes, and Thymus is the major central immune organ and responsible for T cell development and maturation, thymic epithelial cells (TECs) are responsible for the autoimmune tolerance of T lymphocytes, thus TECs is our research objective [3, 4]. The clinical treatment for MS is mainly hormone and immunosuppressive drugs, but it cannot fundamentally remove the cause. Relevant studies have shown that MS may be caused by immune disorders caused by the interaction of genetic factors and environmental factors [5, 6]. Therefore, further study on the etiology and mechanism of MS is of great importance for the radical cure of this disease.

Bioinformatics is a new interdisciplinary subject that combines life science and computer science. It mainly studies the collection, storage, processing, dissemination, analysis and interpretation of biological information. Through the use of biological and informatics technology can process and analyze a large number of complex biological data. Microarray data information analysis technology has been widely used in the study of various diseases to explore the genetic correlation [7, 8]. Microarray analysis can simultaneously capture the expression of tens of thousands of genes to uncover genomic changes associated with the development of disease. At present, a large number of studies [9, 10] have used bioinformatics technology to analyze the differentially expressed genes in the occurrence of diseases, and further study their roles in biological processes, molecular functions and signaling pathways, and elucidate the pathogenesis of diseases, so as to provide theoretical basis for early diagnosis and treatment.

Nicotinamide adenine dinucleotide (NAD+) is a key coenzyme of hydrogen ion transfer in redox reaction, and is widely involved in many physiological activities such as substance metabolism, energy synthesis, gene expression, DNA repair and aging of cells. Studies in the field of ischemia and trauma have also proved that NAD+ is a neuroprotective agent with multiple targets and has potential therapeutic value for a variety of diseases. SIRT1 is an NAD+ dependent protein deacetylase that catalyzes the removal of acetyl groups from a variety of protein substrates. Its physiological effect depends on NAD+. Related studies have shown that SIRT1 can enhance mitochondrial phagocytosis by inhibiting the PI3K/ Akt/mTOR signaling pathway [11]. Then, whether SIRT1 can also exert a protective effect on EAE mice by inhibiting the PI3K/ Akt /mTOR signaling pathway. Few studies have been done. Therefore, this paper intends to use bioinformatics technology to mine the differential genes between multiple sclerosis and normal tissues, and carry out enrichment analysis and pathway analysis. Using public data to validate the role of core genes. In addition, Experimental autoimmune encephalomyelitis (EAE) mice with similar pathological features and clinical symptoms to MS were used as animal models [12]. To investigate whether NAD+ regulates thymus autophagy and immunity in EAE mice through SIRT1.

## MATERIALS AND METHODS

### Bioinformatics analysis

#### Access to public data

Multiple sclerosis related data sets GSE135511 and GSE131279 were searched and downloaded from GEO (GENE EXPRESSION OMNIBUS) database https://www.ncbi.nlm.nih.gov/gds/. Limma package of R language was used to perform quantile standardized preprocessing on GSE135511 and GSE131279 data sets, respectively. Differential gene analysis was performed to obtain the relevant differential genes (|logFC| < 0.5, *p*-value < 0.05), and volcano map and cluster analysis heat map were drawn for the differential genes. The common differential genes of GSE135511 and GSE131279 were screened using the RobustRankAggreg packet of R language (|logFC| < 0.5, *p*-value < 0.05).

#### Functional enrichment analysis

Go (Gene Ontology) enrichment analysis and KEGG (Kyoto Encyclopedia of Genes and Genomes) enrichment analysis were performed on the common differentially expressed Genes obtained from GSE135511 and GSE131279 datasets. David online database tool (https://david.ncifcrf.gov) was used to analyze the relative differentially expressed genes at the three levels of biological process, cell component and molecular function, so as to integrate GO terms and create a biological process network of differentially expressed genes. GOPlot and ggplot2 package were used to plot the GO pathway and KEGG pathway enrichment analysis of differentially expressed genes in R language environment.

#### Protein-protein interaction network analysis

Protein interaction network analysis (PPI) database (https://www.string-db.org/) and STRING database were used to process the differential genes to generate the interaction network. And use cytoHubba of cytoscape to identify key genes.

#### Gene set enrichment analysis

Gene Set Enrichment Analysis (GSEA) tool (http://www.gsea-msigdb.org/) was used for GSEA enrichment analysis of all genes, and GSEA enrichment analysis pathway map was drawn.

#### Statistical analysis of target genes

Two independent samples *t*-test was used to compare the two groups of data. GraphPad Prism software was used for drawing. *P* < 0.05 was considered statistically significant.

### Experimental animals and treatment methods

Forty female C57BL/6 mice at 6–8 weeks were randomly divided into 4 groups: control group (*n* = 10), EAE group (*n* = 10), NAD+ group (*n* = 10) and SIRT1 inhibitor group (*n* = 10). The mice in EAE group, NAD+ group and SIRT1 knockout group were prepared by MOG35-55 polypeptide immunoassay. Mice in both the NAD+ group and the SIRT1 inhibitor group were treated with NAD+ drug and continued to feed for 4 weeks.

### The origin and grouping of experimental cells

The thymus tissues of wild-type C57BL/6 female mice were asepsis removed, ground and filtered into single-cell suspension, and then cultured in DMEM. The thymus tissues were divided into four groups: control group, EAE group, NAD+ group and SIRT1 inhibitor group. The EAE group, NAD+ group and SIRT1 inhibitor group were counted with cell counting plate, and the cells were seeded in 6-well plate after adjusting the concentration, and the cells were stimulated by MOG 35-55 (1 μg/mL) to make EAE model *in vitro*. Both the NAD+ group and the SIRT1 inhibitor group were treated with NAD+.

### Experiment materials and instruments

p-PI3K, p-AKT, p-mTOR and total-PI3K, AKT, mTOR were all purchased from abcam; DMEM, PBS buffer and trypsin were purchased from Hyclone Corporation, USA; Fetal bovine serum from GIBCO, USA; Tissue embedding machine (model: EG11508) and paraffin section model (model: RM2135) were purchased from Leica Company; Western Blot electrophoresis tank (DYCZ-24DN), transfer electrophoresis apparatus (DYY-7B) and thermostat circulator (WD-9412A) were purchased from Beijing Liuyi Biological Technology Co., Ltd; Automatic gel imaging system (model: 8845-S) was purchased from Bio-rad Corporation, USA; The electronic balance (model: MP200A) was purchased from Shanghai Jingke Instrument Factory; The ice making machine (model: SIM-F124) was purchased from Japan 5ANY0 Co., Ltd; Enzyme-label instrument (model: ELX800, Beijing Bio-Tek Company) and other related reagents and instruments.

### Methods of animal experiment

#### Neurological function score of mice in each group

From Day 0 to Day 28, the feeding status and clinical symptoms of each group were observed by the same person at the same time every day (10:00 am). The neurological function deficit of each group was scored by a double-blind method. The neurological function score was scored by Weaver (15 points), and the scoring criteria were as follows: tail: 0 points: no clinical symptoms; 1 point: reduced tail tension or distal paralysis; 2 points: total paralysis of the tail; Extremities: 0 points: no symptoms; 1 points: Unsteady gait; 2 points: limb paralysis, dragging phenomenon when walking; 3 points: the limbs showed total paralysis, valgus phenomenon appeared when walking.

#### The autophagy degree of LC-3A/B was observed by immunofluorescence in thymus

Tissue was fixed with 4% paraformaldehyde for 15 min, then soaked with PBS for 3 times, each time for 3 min. 0.2%Triton X-100 permeable at room temperature for 20 min; Soak and wash PBS for 3 times, 3 min each time. Blot the PBS with absorbent paper, drop normal goat serum on the slide, and seal it at room temperature for 30 min. Blot off the blocking solution with absorbent paper, add enough diluted primary antibody to each slide and put it into a wet box for overnight incubation at 4°C. Fluorescent secondary antibody was added and soaked with PBS for 3 times, 3 min each time. After the excess liquid on the slider was dried with absorbent paper, diluted fluorescent secondary antibody was added and incubated at room temperature in the wet box for 1 h. PBS soaked slices for 3 times, 3 min each time. DAPI was dropped and incubated for 5 min in the dark. The specimens were dyed and the redundant DAPI was washed off with PBS. Blot the liquid on the sliver with absorbent paper, seal the sliver with the sealing solution containing anti-fluorescence quenching agent, observe and collect the image under the fluorescence microscope.

#### The expressions of p-PI3K, p-AKT, p-mTOR, P62, Beclin1, LC-3A/B and SIRT1 were detected by WB

The above tissues were ground to extract proteins, and then WB detection was performed. After protein separation by polyacrylamide gel electrophoresis (SDS-PAGE), the membrane was transferred to PVDF membrane, sealed with 5% skimmed milk powder for 2 hours, and incubated overnight with primary antibody in a refrigerator at 4°C. After fully washing PBST, corresponding secondary antibody (AB97080) was added and incubated at room temperature for 1h. ECL color was developed in chemiluminescence gel imaging system, gray scanning analysis was performed with IPP 6.0 software, and GAPDH was used as internal reference to correct the expression level of target protein.

### Methods of cell experiment

#### The autophagy degree of LC-3A/B was observed by immunofluorescence

Place the slides into a 24-well plate and add a certain density of the above-mentioned cultured cells, overnight. The cell slides were placed into a 35 mm cell culture dish and soaked with PBS for 3 times, 3 min each time. The slides were fixed with 4% paraformaldehyde for 15 min, then soaked with PBS for 3 times, 3 min each time. 0.2%Triton X-100 permeable at room temperature for 20 min; Soak and wash PBS for 3 times, 3 min each time. Blot the PBS with absorbent paper, drop normal goat serum on the slide, and seal it at room temperature for 30 min. Blot off the blocking solution with absorbent paper, add enough diluted primary antibody to each slide and put it into a wet box for overnight incubation at 4°C. Fluorescent secondary antibody was added and soaked with PBS for 3 times, 3 min each time. After the excess liquid on the slider was dried with absorbent paper, diluted fluorescent secondary antibody was added and incubated at room temperature in the wet box for 1 h. PBS soaked slices for 3 times, 3 min each time. DAPI was dropped and incubated for 5 min in the dark. The specimens were dyed and the redundant DAPI was washed off with PBS. Blot the liquid on the sliver with absorbent paper, seal the sliver with the sealing solution containing anti-fluorescence quenching agent, observe and collect the image under the fluorescence microscope.

#### The expressions of p-PI3K, p-AKT, p-mTOR, P62, Beclin1, LC-3A/B and SIRT1 were detected by WB

The above groups of cells were ground to extract proteins for WB detection. After protein separation by polyacrylamide gel electrophoresis (SDS-PAGE), the membrane was transferred to PVDF membrane, sealed with 5% skimmed milk powder for 2 hours, and incubated overnight with primary antibody in a refrigerator at 4°C. After fully washing PBST, corresponding secondary antibody (AB97080) was added and incubated at room temperature for 1 h. ECL color was developed in chemiluminescence gel imaging system, gray scanning analysis was performed with IPP 6.0 software, and GAPDH was used as internal reference to correct the expression level of target protein.

## RESULTS

### Bioinformatics analysis

#### Screening of DEGs

**Figure 1:**
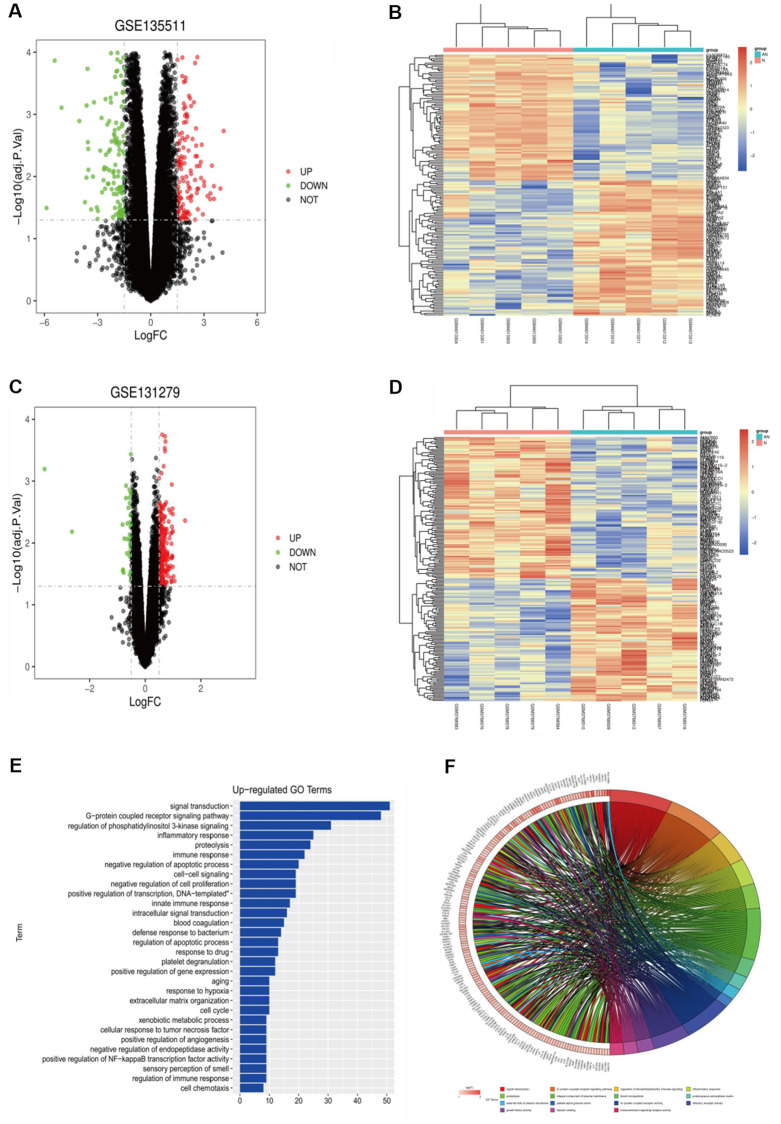
(**A**) GSE135511 differential gene volcano map. (**B**) GSE135511 heat map of differential gene cluster analysis. (**C**) GSE131279 differential gene volcano map. (**D**) GSE131279 heat map of differential gene cluster analysis. (**E**, **F**) GO enrichment analysis of the up-regulated pathway.

The DEGs, related with multiple sclerosis, included 293 up-regulated genes and 82 down-regulated genes. Volcanic maps (Figure 1A) and cluster analysis heat maps (Figure 1B) were drawn for the differential genes. The Limma packet of R language was used to perform quantile standardized preprocessing on the data set GSE131279, and differential gene analysis was performed to obtain relevant differential genes (|logFC| < 0.5, *p*-value < 0.05), including 30 up-regulated genes and 67 down-regulated genes. Volcanic maps (Figure 1C) and cluster analysis heat maps (Figure 1D) were drawn for the differential genes. The common differential genes of GSE135511 and GSE131279 were screened using the RobustRankAggreg package of R language (|logFC| < 0.5, *p*-value < 0.05), including 95 up-regulated genes and 89 down-regulated genes.

#### Functional enrichment analysis

**Figure 2:**
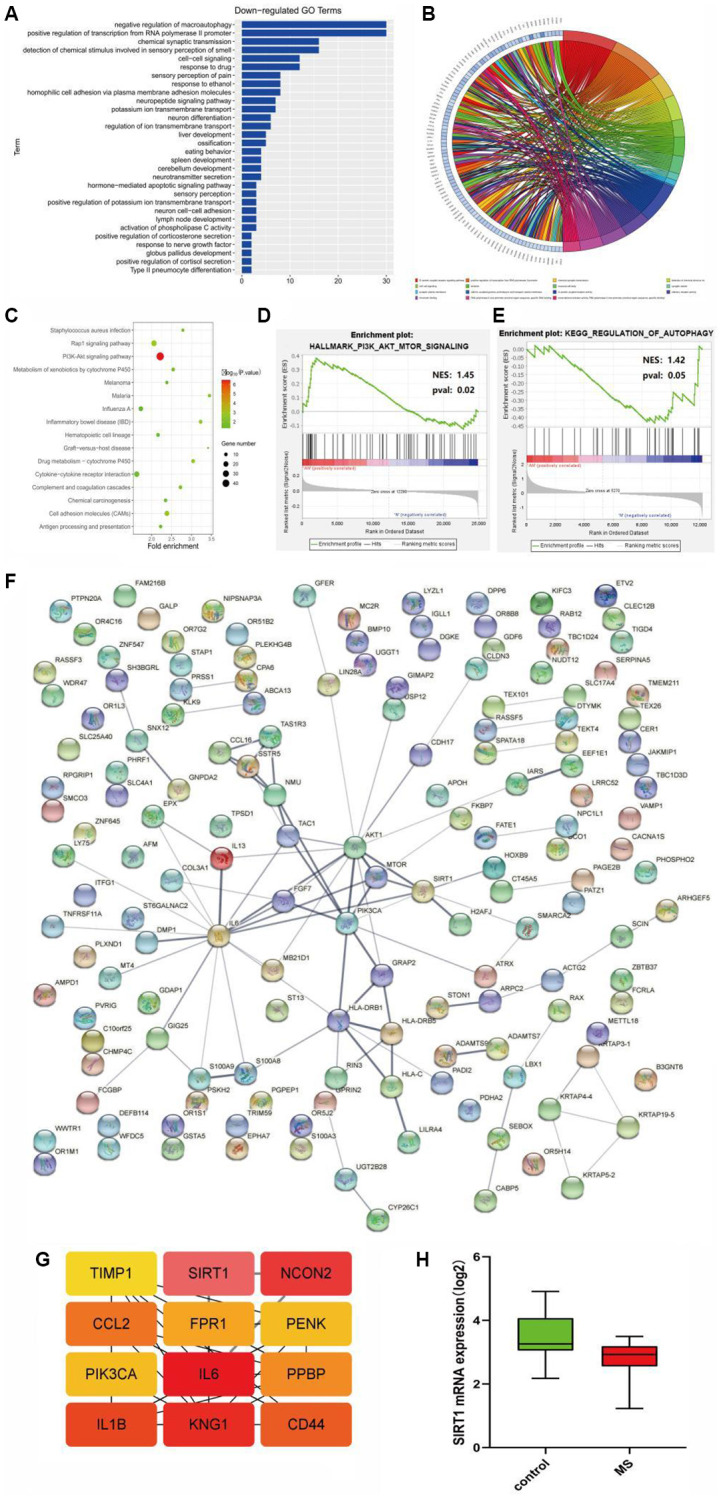
(**A**, **B**) GO enrichment analysis of the down-regulated pathway. (**C**) KEGG pathway enrichment analysis. (**D**, **E**) GSEA gene enrichment analysis. (**F**) The protein-protein interactive network. (**G**) The hub gene network. (**H**) The expression analysis of SIRT1.

GO enrichment analysis was performed using the online tool DAVID, and the up-regulation pathway diagram of GO was drawn using R language (Figure 1E, 1F), including signal transduction, G−protein coupled receptor signaling pathway, regulation of phosphatidylinositol 3-kinase signaling and other pathways. GO down-regulation pathway (Figure 2A, 2B), includes negative regulation of macroautophagy, positive regulation of transcription from RNA polymerase II promoter, chemical synaptic transmission, etc. KEGG pathway was analyzed by using DEGs and KEGG pathway was plotted, which was enriched in the PI3K-AKT signaling pathway, RAP1 signaling pathway and other pathways (Figure 2C). In addition, GSEA gene enrichment analysis showed that it was enriched in PI3K-AKT-mTOR and autophagy pathways (Figure 2D, 2E).

#### Protein-protein interaction network analysis

PPI network analysis was performed on the common DEGs, and PPI network was established based on the STRING database (Figure 2F), and hub genes were identified using cytohubba of cytoscape: TIMP1, SIRT1, NCON2, CCl2, FPR1, PENK, PIK3CA, IL6, PPBP, IL1B, Kng1, CD44 (Figure 2G).

#### Statistical analysis of target genes

The content of SIRT1 in different groups was analyzed by statistical methods, and it could be seen that the expression of SIRT1 was low in the disease group and high in the control group (Figure 2H).

### Animal experiment results

#### Neurological function score of mice in each group

**Figure 3:**
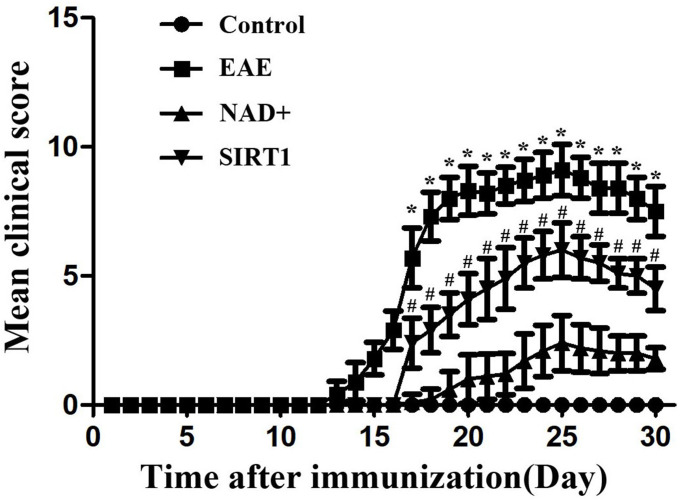
Neurological function score of mice in each group.

The mice in the control group did not show clinical symptoms of neurological impairment, and the activity and response of the mice were not abnormal. The EAE group, the NAD+ group and the SIRT1 inhibitor group showed different degrees of morbidity successively after modeling. After 27 days of modeling success, the clinical manifestations of EAE mice reached the peak of morbidity, with the most serious symptoms of neurological impairment, such as weakness and paralysis of both hind limbs. The following groups were SIRT1 inhibitor and NAD+ group (Figure 3).

#### The autophagy degree of LC-3A/B at the level of mice was observed by immunofluorescence in thymus

**Figure 4:**
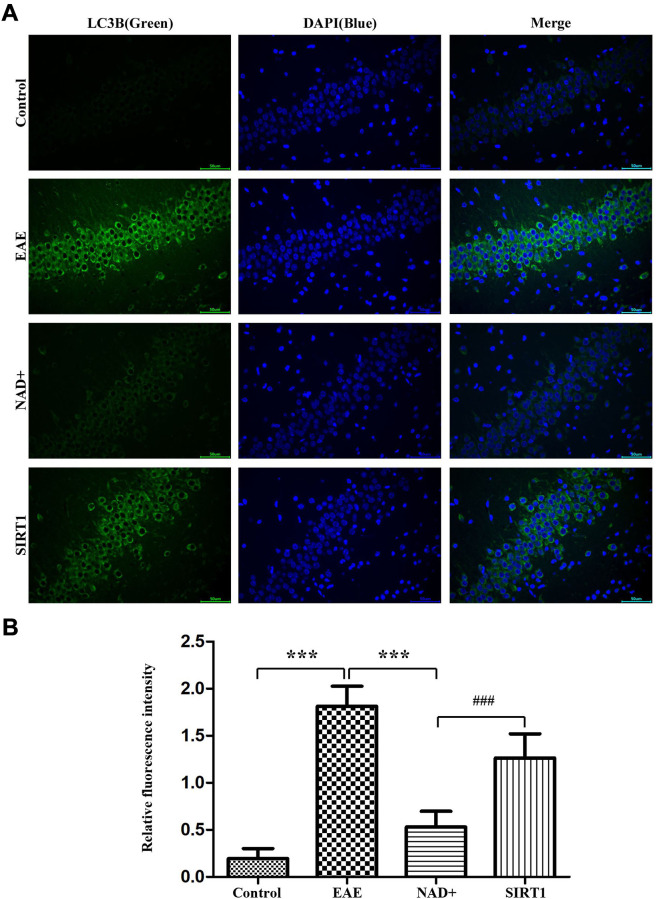
**Autophagy degree of LC-3A/B at the level of mice.** (**A**) Under fluorescence microscope, immunofluorescence staining was used to present the expression of LC-3A/B at the level of mice. (**B**) Column diagram manifesting the relative fluorescence intensity in the different group at the level of mice.

Autophagy is the pivotal process for maintaining homeostasis of organism. Under fluorescence microscope, immunofluorescence staining were carried on in the thymus, our results showed that the fluorescence intensity of LC-3A/B in EAE group was the highest, followed by NAD+ + SIRT1 inhibitor group, NAD+ group, and control group (Figure 4). As the LC-3A/B is the typical marker protein for autophagy, and suppression of autophagy is the important driving factors for the disease of EAE, thus in these results deficiency/absent of autophagy appeared in EAE mice’ brain and the NAD+ significantly increased the incidence of autophagy, SIRT1 inhibitor SIRT-IN-3 alleviated the NAD+’ effects. Our results indicated the NAD+’ protective roles for EAE were dependent, at least in partly, on SIRT1 signal.

#### The expressions of p-PI3K, p-AKT, p-mTOR, P62, Beclin1, LC-3A/B and SIRT1 were detected by WB

**Figure 5:**
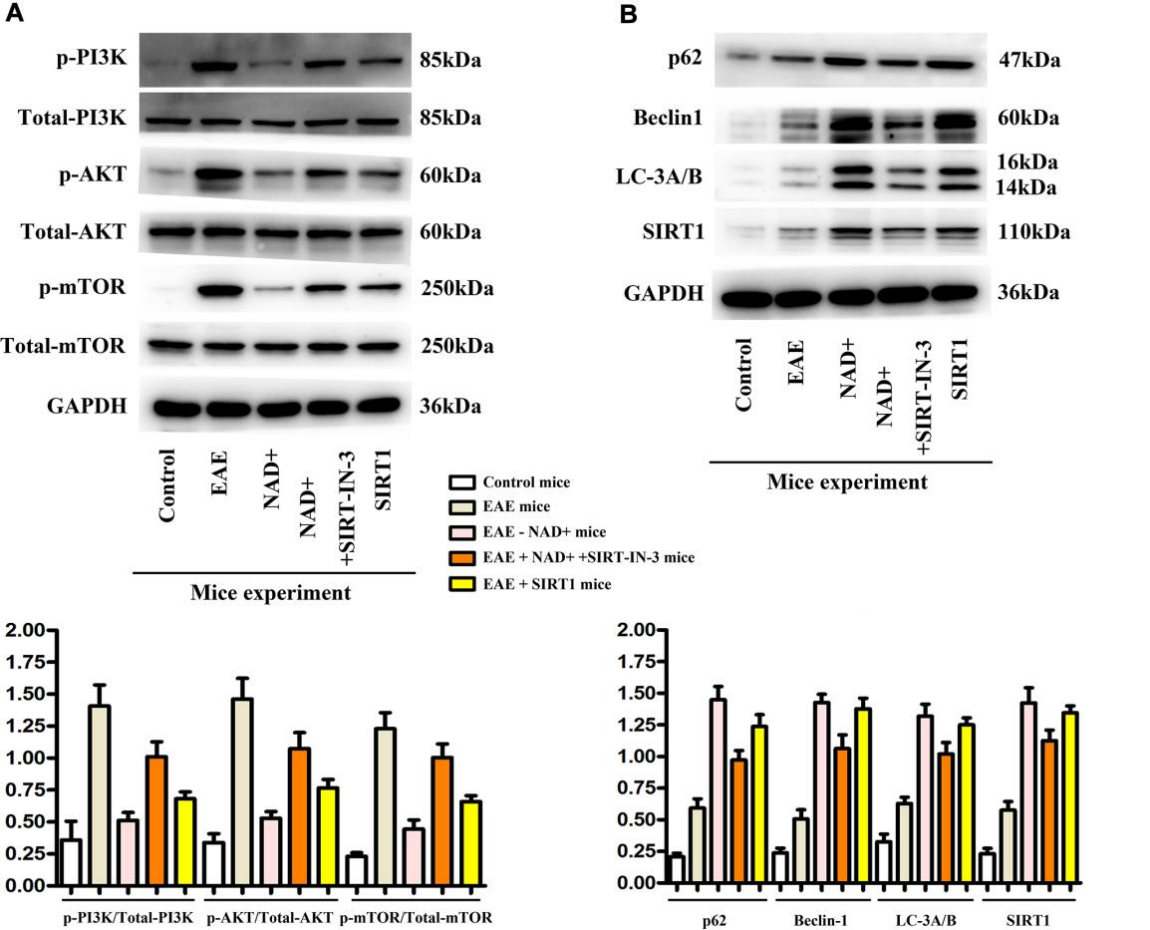
**Expression of p-PI3K, p-AKT, p-mTOR, P62, Beclin1, LC-3A/B and SIRT1 in tissues.** (**A**) Western blotting showed that expressions of p-PI3K, p-Akt and p-mTOR were the highest in the EAE group at the level of tissues. (**B**) P62, Beclin1, LC-3b and SIRT1 were the up-regulated in the NAD+ group, followed by the NAD+ + SIRT-IN-3 group, the EAE group and the control group at the level of tissues by western blotting.

In our mice experiments, the expressions of p-PI3K, p-AKT, p-mTOR, P62, Beclin1, LC-3A/B and SIRT1 were observed in the chemiluminescence gel imaging system. It was found that the expressions of P62, Beclin1, LC-3A/B and SIRT1 were the highest in the NAD+ group, followed by the NAD+ + SIRT-IN-3 group, the EAE group and the control group, while the expressions of p-PI3K, p-Akt and p-mTOR were the highest in the EAE group. The following were NAD+ + SIRT-IN-3 group, NAD+ group, control group and the SIRT1 showed the similar tendency as NAD+ group (Figure 5). Autophagy effectively removed damaged organelles and suppressed the incidence of apoptosis, preserving the integrity of brain in the disease of EAE. The activation or phosphorylated-PI3K/AKT/mTOR signals are key and primary regulatory proteins for inhibition of autophagy, thereby, the inhibition of the PI3K could opportunely and competitively induced the incidence of autophagy. NAD+ effectively decreased phosphorylated PI3K, AKT, mTOR, vs EAE group, and SIRT-IN-3 relieved NAD+’ roles. SIRT1 is the key suppressor for PI3K signals, and combined with these results, NAD+ alleviated PI3K and increased autophagy may by increasing SIRT1 expression in EAE diseases.

### Cell experiment results

#### The autophagy degree of LC-3A/B at the cellular level was observed by immunofluorescence

**Figure 6:**
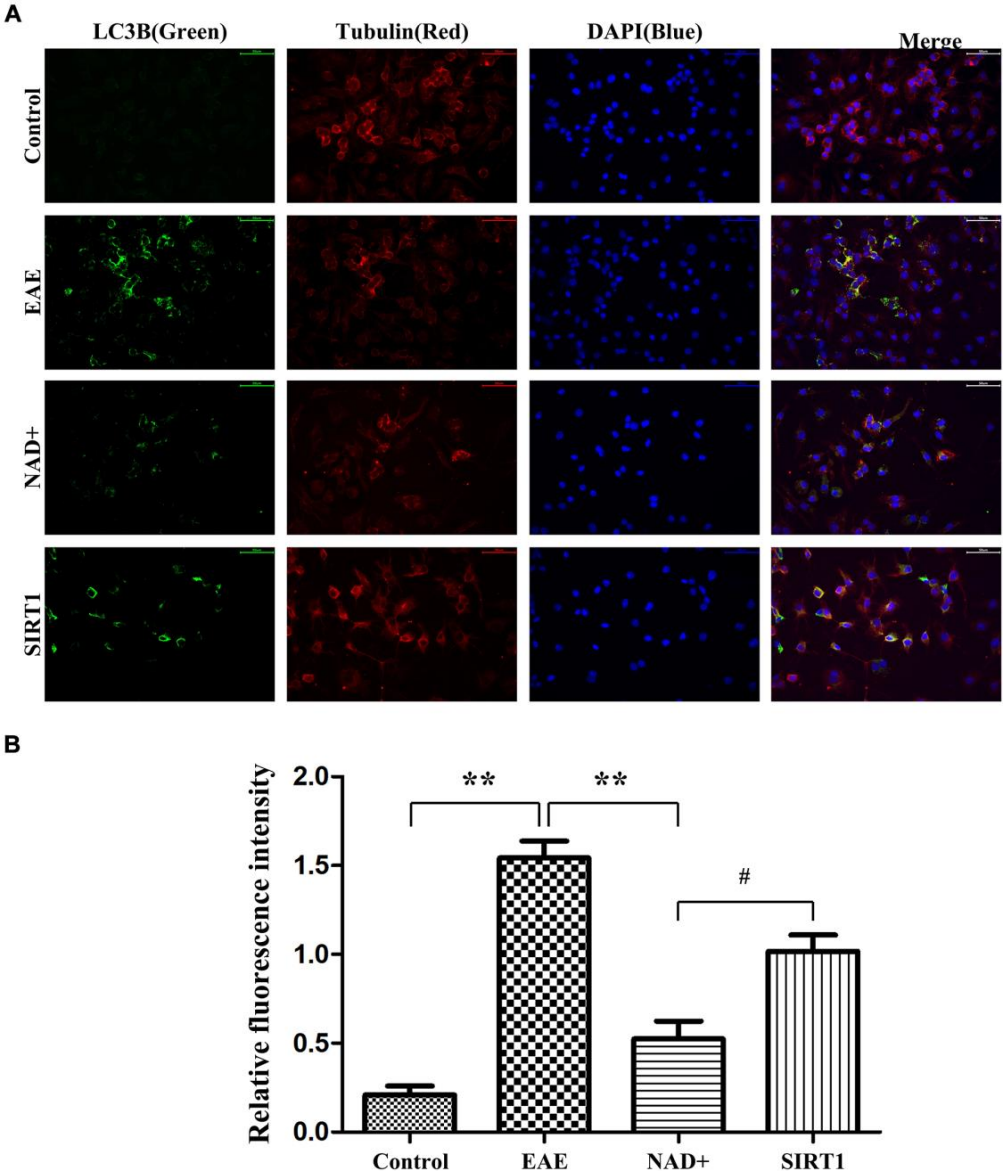
**Autophagy degree of LC-3A/B at the cellular level.** (**A**) Under fluorescence microscope, immunofluorescence staining was used to present the expression of LC-3A/B at the cellular level. (**B**) Column diagram manifesting the relative fluorescence intensity in the different group at the cellular level.

Under fluorescence microscope, immunofluorescence staining showed that the fluorescence intensity of LC-3A/B in EAE group was the highest, followed by SIRT1 inhibitor group, NAD+ group, and control group (Figure 6).

#### The expressions of p-PI3K, p-AKT, p-mTOR, P62, Beclin1, LC-3A/B and SIRT1 were detected by WB

**Figure 7:**
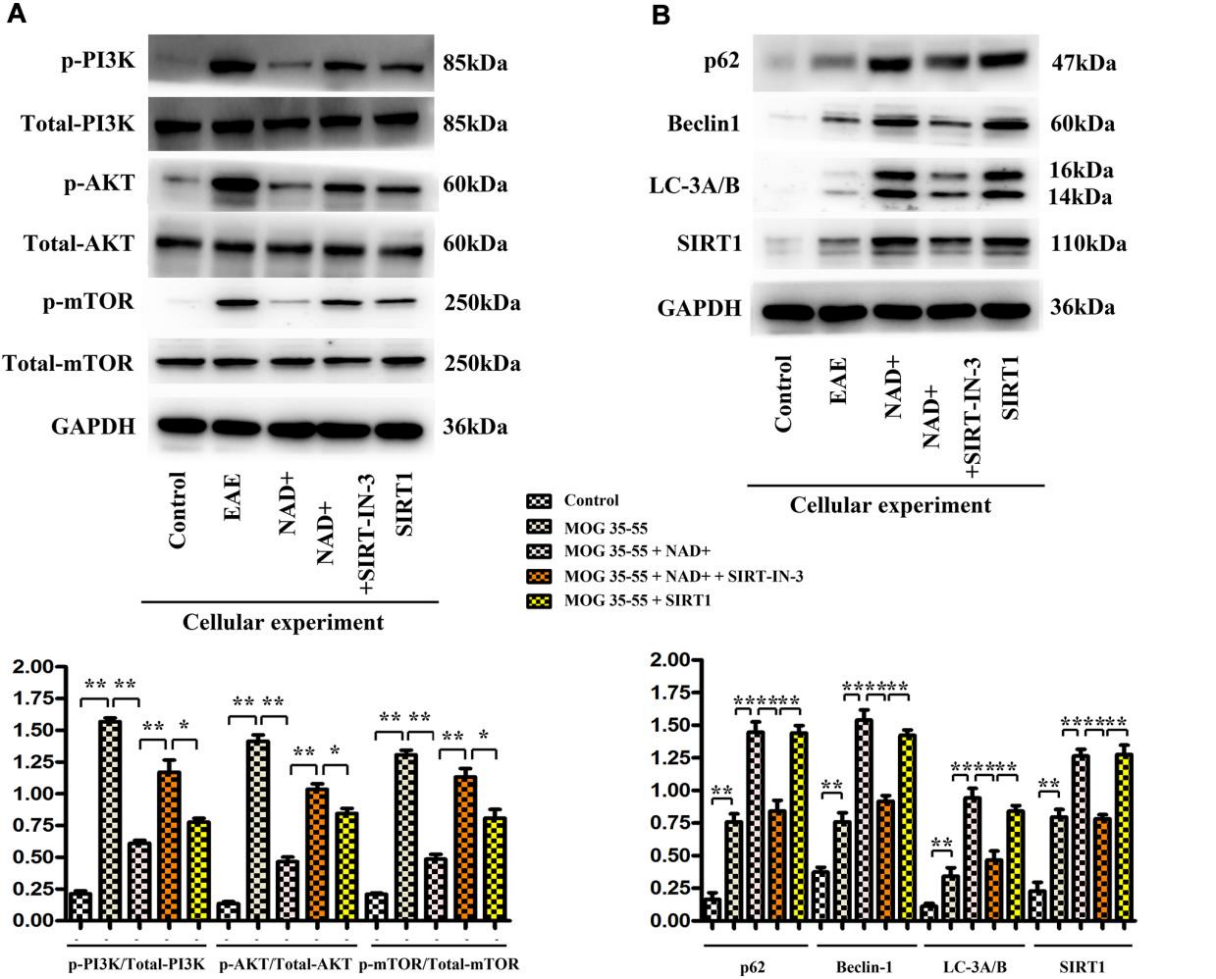
**The expression of p-PI3K, p-AKT, p-mTOR, P62, Beclin1, LC-3A/B and SIRT1 in cells.** (**A**) Western blotting showed that expressions of p-PI3K, p-Akt and p-mTOR were the highest in the EAE group at the level of cells. (**B**) P62, Beclin1, LC-3b and SIRT1 were the up-regulated **www.aging-us.com** 14 AGING in the NAD+ group, followed by the SIRT1 inhibitor group, the EAE group and the control group at the level of cells by western blotting.

The expressions of p-PI3K, p-AKT, p-mTOR, P62, Beclin1, LC-3A/B and SIRT1 were observed in the chemiluminescence gel imaging system. It was found that the expressions of P62, Beclin1, LC-3A/B and SIRT1 were the highest in the NAD+ group, followed by the SIRT1 inhibitor group, the EAE group and the control group, while the expressions of p-PI3K, p-Akt and p-mTOR were the highest in the EAE group. The following were SIRT1 inhibitor group, NAD+ group, and control group (Figure 7).

## DISCUSSION

MS is a chronic inflammatory autoimmune disease of the central nervous system mediated by T cells. Its main pathological feature is inflammatory demyelinating changes of the central nervous system [13]. This disease tends to occur in young and middle-aged people [14], and is mostly seen in women. At present, relevant researchers generally accept that the pathogenesis of MS is an autoimmune response mediated by T cells, which mainly targets the myelin antigens of the central nervous system [15]. Because its etiology and pathogenesis are not very clear, it cannot cure its disease fundamentally. Among them, EAE animal model is internationally recognized as the classic animal model of MS, which is widely used in basic research of MS [16]. Thymus is the major central immune organ and responsible for T cell development and maturation, T cells recognize our own antigens in thymic epithelial cells (TECs), which result in the clonal deletion or clonal non-response of autoreactive T cells and lead to autoimmune tolerance [3, 4]. Therefore, in this study, EAE mice were used as the animal experimental model of MS to explore the effect of NAD+ on the thymus autophagy mechanism of EAE mice through SIRT1. Through the bioinformatics, KEGG enrichment analysis manifested that the PI3K-AKT-mTOR was one significant pathway in the occurrence and development of multiple sclerosis. In addition, GSEA gene enrichment analysis also demonstrated the above results. Furthermore, through the cytohubba SIRT1 was identified as the hub gene of the multiple sclerosis.

Silence Information Regulator 1 (SIRT1) is a highly conserved NAD+ dependent histone deacetylase, which is closely related to the formation of autophagy [17, 18]. The endogenous substrates of SIRT1 are very rich, including p53, p300, NF-κB, c-Jun, etc. Each deacetylation of SIRT1 hydrolyzes one nicotinamide adenine dinucleotide (NAD+). Therefore, SIRT1 is also called NAD+ dependent protein deacetylase [19]. It has anti-inflammatory, anti-oxidative and anti-apoptotic effects, and its biological function mainly depends on its deacetylase activity [20]. Zhu et al. [21] confirmed through mouse model and *in vitro* experiments that SIRT1 can inhibit mTOR phosphorylation, inhibit inflammation and fibrosis, and achieve the effect of improving scleroderma, suggesting that SIRT1 has a certain therapeutic effect on systemic sclerosis. Studies on patients with multiple sclerosis showed that SIRT1 increased in damaged brain tissue and SIRT1 decreased in peripheral blood monocytes when multiple sclerosis recurred, suggesting a certain relationship between SIRT1 and the pathogenesis of multiple sclerosis [22]. Li et al. [23] showed that the serum SIRT1 level was decreased and the level of inflammatory chemokine CCL20 was increased in patients with multiple sclerosis, suggesting that SIRT1 may inhibit the expression of CCL20 through the NF-κB pathway, and the lack of SIRT1 may be involved in the pathogenesis of multiple sclerosis.

SIRT1 deacetylates autophagy-related proteins (such as Beclin-1 and LC3) can promote autophagy. Beclin-1 expression level is related to the acetylation of lysine residues. Its acetylation inhibits the formation of autophagosomes [24]. Deacetylation of Beclin-1 lysine residue by SIRT1 affects the formation of autophagosomes and subsequent biological effects, suggesting that Beclin-1 is a deacetylation target of SIRT1. SIRT1-mediated autophagy plays an important role in nerve cell injury, myocardial cell remodeling and other diseases [25, 26]. However, there are few studies on whether it also plays such a regulatory role in thymus autophagy. Therefore, by stimulating the inhibitor of SIRT1 with NAD+ treatment, we found that the expression of autophagy-related proteins (P62, Beclin1, and LC-3A/B) was significantly increased after NAD+ treatment, consistent with decreased expression of these proteins in NAD+ +SIRT-IN-3 group *in vivo* (Figure 5) and *in vitro* experiments (Figure 7). Cell experiments further confirmed that the expression of autophagy protein in thymus epithelial cells treated with NAD+ drugs was consistent with animal experiments. Therefore, we believe that the activation of SIRT1 can also activate the autophagy level of the thymus and play a protective role. So, how does SIRT1 activate autophagy in the thymus? Related studies [11] have shown that the activation of SIRT1 can inhibit the activation of PI3K/ AKT /mTOR signaling pathway, thereby promoting autophagy of nerve cells and finally playing a protective role in nerve cells. Further studies [27] showed that the expression of proteins related to PI3K/ AKT /mTOR signaling pathway in SIRT1 knockout macrophages was significantly increased, thereby inhibiting macrophage autophagy and eventually causing the formation of atherosclerosis. Therefore, based on the studies of the above scholars, this study inferred that SIRT1 could also exert mouse thymus autophagy by inhibiting the activation of PI3K/ AKT /mTOR signaling pathway, and ultimately inhibit the further deterioration of EAE mice. Considering the closely connection between the PI3K/AKT/mTOR and autophagy proteins as well as the bioinformatics results, the associated activation and expression were detected in our study in EAE, NAD+ and NAD+ +SIRT-IN-3 groups *in vivo* and *in vitro* experiments by western blot. It was found that PI3K/AKT/mTOR signaling proteins were significantly decreased in animal and cell experiments after the administration of NAD+, the inhibitor of SIRT1, while autophagy proteins (P62, Beclin1, LC-3A/B) were significantly decreased, these results confirmed the NAD+ could inhibited the PI3K/AKT activation and resulted in the unhindered autophagy, and NAD+’ effects were weakened by the specific inhibitor of SIRT1: SIRT-IN-3. After the administration of SIRT1 inhibitor, PI3K/ AKT/mTOR signaling proteins were significantly increased in animal and cell experiments, while autophagy proteins (P62, Beclin1, LC-3A/B) were significantly decreased. Therefore, this study suggests that activated SIRT1 can inhibit the PI3K/AKT/mTOR signaling pathway, and thus promote the occurrence of autophagy in the thymus. The thymus is the main site of regulating central immunity and the main site of T cell differentiation. Autophagy, as an important process to maintain homeostasis in the body, is essential for positive and negative selection of T cells in the thymus [28, 29]. When the specific selection of T cells is abnormal, it can cause severe colitis and multiple organ inflammation [30]. In this study, HE staining and neurological function score of mice showed that the spinal cord inflammatory injury of EAE mice was significantly alleviated and the neurological function recovery state was significantly improved after treatment with NAD+, the activator of SIRT1, suggesting that NAD+, the activator of SIRT1, could play a protective role in the neurological function of EAE mice by promoting thymic autophagy.

In conclusion, we confirmed the regulation of SIRT1 on autophagy in the EAE mouse model. NAD+ promotes autophagy of thymus epithelial cells by stimulating the expression of SIRT1. Then, the autoimmune state of EAE mice was inhibited, so that EAE mice were protected from sustained damage. Considering that SIRT1 plays an important role in the occurrence and progression of EAE mice model, the above results provide a possible target for the future clinical treatment of MS.
